# Gankyrin Is Frequently Overexpressed in Cervical High Grade Disease and Is Associated with Cervical Carcinogenesis and Metastasis

**DOI:** 10.1371/journal.pone.0095043

**Published:** 2014-04-21

**Authors:** Yuan Liu, Jiawen Zhang, Wenyan Qian, Yu Dong, Yongbin Yang, Zhiqiang Liu, Youji Feng, Ding Ma, Zhenbo Zhang, Sufang Wu

**Affiliations:** 1 Department of Obstetrics and Gynecology, Shanghai First People’s Hospital, Shanghai Jiaotong University, Shanghai, China; 2 Department of Obstetrics and Gynecology, Shanghai Tenth People’s Hospital, Shanghai Tongji University, Shanghai, China; 3 Department of Gynecology and Obstetrics, Kunshan Hospital of Traditional Chinese Medicine Affiliated to Nanjing University of Chinese Medicine, Jiangsu, China; 4 Division of Cancer Medicine, Department of Lymphoma and Myeloma, Center for Cancer Immunology Research, The University of Texas M. D. Anderson Cancer Center, Houston, Texas, United States of America; 5 Cancer Biology Medical Center, Tongji Hospital, Tongji Medical College, Huazhong University of Science and Technology, Wuhan, China; University of Navarra, Spain

## Abstract

Our previous studies have showed that Gankyrin expression is correlated with a malignant phenotype in endometrial carcinoma. Here, we investigated the possible role of Gankyrin in cervical disease. The increasing protein level of Gankyrin was observed in high-grade cervical intraepithelial neoplasia and carcinoma compared with benign cervical tissues and low-grade cervical intraepithelial neoplasia. In para-carcinoma tissues, it was found interestingly that there was no lymph node metastasis when nuclei Gankyrin was positively expressed, but lymph node metastasis rate was 30% (6/20) when nuclei Gankyrin was negatively expressed. In vitro, the transfection of Gankyrin resulted in markedly up-regulating of Vimentin, β-catenin and Twist2, as well as down-regulating of E-cadherin in cervical carcinoma cells. Our results suggested that Gankyrin may be functional in cervical carcinogenesis and metastasis.

## Introduction

Cervical carcinoma is the third leading cause of cancer-related death in women worldwide. The American Cancer Society estimates that 12,340 women will be diagnosed with invasive cervical cancer, and 4,030 women will die from the disease in 2013 [1]. There are more than 130 thousand cases of cervical cancer newly found in China every year, which accounts for 1/4 of all the cases of cervical cancer around the world, and more than 30 thousand of them are being died. As we all know, more than 99% of cervical carcinomas are positive for high-risk human papillomaviruses (HPVs) [2]. HPV E6 and E7 function as the dominant oncoproteins of ‘high-risk’ HPVs by altering the function of critical cellular proteins, such as p53 and retinoblastoma tumor suppressor protein (Rb) [3]. Despite extensive studies focused on the mechanisms of HPV-induced cervical carcinogenesis, the downstream signaling events mediated by other important factors remain obscure.

We previously showed the elevated-expression pattern of Gankyrin in endometrial carcinomas and confirmed its role in endometrial cancer development [4], however, whether it also involves in cervical carcinoma occurrence remains unclarified. Gankyrin has emerged as a critical oncoprotein found overexpressed early in hepatocarcinoma, with potent function of regulating cell cycle and anti-apoptosis [4]. Previous findings showed that Gankyrin was negatively or weakly expressed in some normal tissues, but its protein level was increased in a range of tumor tissues, including hepatocellular carcinoma, esophageal squamous cell carcinoma, breast carcinoma and endometrial carcinoma [4]–[7]. An ample supply of evidence demonstrated that Gankyrin could not only modulate the phosphorylation of Rb by CDK4, but also furtherance the ubiquitylation of p53 by the RING ubiquitin ligase MDM2 [8]. Therefore,there are intriguingly considerable similarities of the classic carcinogenic mechanisms found between Gankyrin and HPV pathway.

It is well demonstrated that tumor cells change morphology, acquire migratory and invasive capacity through the process of epithelial–mesenchymal transition (EMT). The decreasing expression of E-cadherin and the increasing expression of Vimentin are reported to be essential hallmarks of the EMT process [9]. Though the mechanisms of Gankyrin strikingly resemble the pathways of HPV oncoprotein, Gankyrin expression pattern in cervical lesion tissues and its role in cervical carcinogenesis and metastasis, especially the EMT process,have not been reported before. In the present study, we investigated the protein level of Gankyrin in normal cervical, cervical intraepithelial neoplasia (CIN) and cervical carcinoma tissues by immunohistochemistry (IHC). We have also investigated the mechanisms of the action of Gankyrin in cervical carcinogenesis and metastasis.

## Materials and Methods

### 1 Ethics

This study was conducted according to the tenets of the Declaration of Helsinki for the use of human subjects and all the species for immunohistochemical staining was approved by Ethics Committee of Shanghai First People’s Hospital, Shanghai Jiaotong University, Shanghai, China (Permit Number:2012K038) and informed consents were obtained from all patients.

### 2 Tissue Samples

Archived cervical specimens representing a wide range of cervical disease processes were selected for analysis from the case files of the Department of Obstetrics and Gynecology from Shanghai Jiao Tong University, Affiliated First People’s Hospital between February 2009 and August 2012. 6 cases of normal cervical tissues, 30 cases of cervical intraepithelial neoplasia tissues (including 11 CIN I cases, 19 CIN II-III cases), 40 cases of cervical squamous cell carcinoma tissues (SCC). 30 pairs of cervical squamous cell carcinoma tissues and tumor adjacent tissues were also included. Pathological diagnoses of cervical samples were managed by two experienced gynecologic pathologists (J.T. Xu and Z.L.Chen) basing on the World Health Organization (WHO) classification in a double-blinded manner.

### 3 Immunohistochemical Staining

IHC analysis of Gankyrin protein expression was performed as previously described [4]. The sections were incubated with rabbit anti-human Gankyrin antibody (diluted to 1∶100; Sigma, St. Louis, MO, USA), and omitted primary antibodies served as negative controls. Expression of Gankyrin protein was assessed by a semiquantitative method: the sections were assessed for the intensity of the staining (0–3) and the percentage of positively stained cells (0–3). Index of Gankyrin expression was calculated as percentage×intensity of the staining. Therefore, score 0 presents negative (−), 1–3 serves as weak positive (+), 4–6 as positive (++), and 7–9 as strong positive (+++).

### 4 Cell Lines and Cell Culture

Human cervical carcinoma lines Siha and Hela purchased from ATCC, were maintained in Dulbecco’s modified Eagle’s medium (DMEM) F-12 1:1 medium (GIBCO) with 10% fetal bovine serum (FBS; GIBCO), 100 U/ml penicillin, sodium pyruvate and L-glutamine in a humidified atmosphere of 5% CO_2_ at 37°C.

### 5 Plasmid Transfection and Small Interfering RNA

The pMKITneo-hGankyrin plasmid and pMKITneo empty vector were generous gifts from Dr Jun Fujita (Department of Clinical Molecular Biology, Graduate School of Medicine, Kyoto University, Kyoto, Japan) and the Gankyrin small interfering RNA (siRNA) was purchased from Shanghai GenePharma Co., Lt. The acute siRNA and plasmid transfections ware performed as previously described [10].

### 6 Western Blot Analysis

Western blots were performed as previously described [11]. Briefly, as soon as the harvested cells were lysed and the supernatant was collected, 60 µg protein was loaded onto SDS-PAGE and transferred to polyvinylidene fluoride membranes (PVDF). Membranes were blocked with 5% skimmed milk for 1 hour and incubated overnight with one of the following primary antibodies: anti-PSMD10 (diluted at 1∶1,000; Sigma, St. Louis, MO, USA), anti-Twist2 (diluted at 1∶200; Abcam, Cambridge, UK), anti-Vimentin (diluted at 1∶500; Cell Signaling Technology, Beverley, MA, USA), anti-β-catenin (diluted at 1∶500; Cell Signaling Technology), anti-E-cadherin (diluted at 1∶500; Cell Signaling Technology) and anti-cyclin D1(diluted at 1∶500; Cell Signaling Technology) rabbit monoclonal antibody, followed by 1 hour of incubation with the appropriate secondary antibody (1∶5,000). The anti-GAPDH (Epitomics) rabbit monoclonal antibody was diluted to 1∶1,000 for use as a sample loading control.

### 7 Cell Counting Kit-8 (CCK-8) Assay

HeLa and SiHa cells were plated in 96-well plates (2,000 cells per well), incubated for 24 hours, followed by serum-free medium for another 24 hours. Cells were transiently transfected with Gankyrin siRNA or the pMKIT-Gankyrin plasmid to investigate their roles in cell proliferation. After 72 hours incubated, CCK-8 solution (Signalway Antibody Co., Ltd. Maryland, USA) was added. After 2 hours of incubation at 37°C, the absorbance was measured at 450 nm with a GENios multifunction reader (Tecan, Zurich, Switzerland).

### 8 Invasion Assay

For invasion assays, cells were seeded on top of (100 cell/chamber) BioCoat Matrigel invasion chambers (BD Biosciences). Medium was supplemented with 2% heat-inactivated FBS in the upper chamber, and the lower chamber was filled with 20% FBS used as chemo-attractant. Cells that invade through the Matrigel-coated membrane after 24 hours were fixed with paraformaldehyde for 20 minutes, followed by staining with crystal violet for 15 minutes and washed seven times with PBS. The non-invading cells were removed from the upper surface of the membrane by scrubbing with a cotton-tipped swab. Five fields for each chamber were photographed using a digital camera mounted on an inverted microscope (magnification,×100) and the invading cells were counted in each field.

### 9 Statistical Analysis

The statistical significance of the difference of Gankyrin expression in the IHC staining in cervical tissues was calculated by the Chi-squared test. The difference in various protein levels, cell proliferation and invasion between groups was analyzed by the Student’s t-test. A two-sided test with *p*<0.05 was considered statistically significant. All statistical analyses were performed using SAS Release 8.02 (SAS Institute Inc., Cary, NC, USA).

## Results

### 1 Gankyrin Protein Expression in Human Cervical Tissues

**Figure 1:**
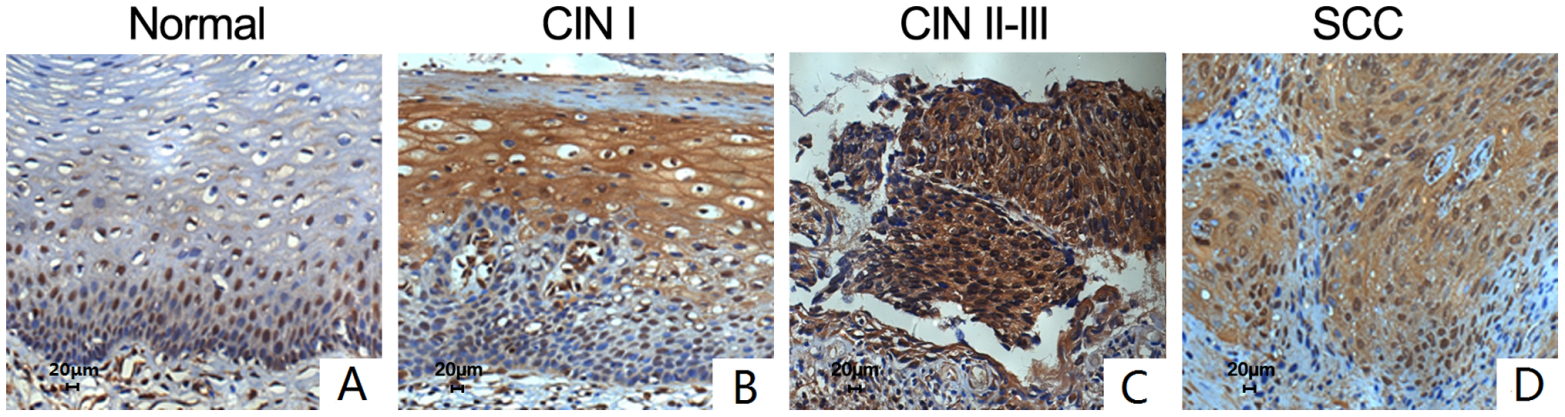
The expression of Gankyrin in all grades of cervical disease by immunohistochemistry staining assay (×200). A. The expression of Gankyrin in normal cervical squamous epithelial; B. The expression of Gankyrin in CIN I; C. The expression of Gankyrin in CIN II–III; D. The expression of Gankyrin in SCC.

The expression localization of Gankyrin was located both in cytoplasm and nucleus (Figure.1). Compared with normal cervix tissues, CIN II–III and cervical carcinoma tissues had stronger positive IHC staining of Gankyrin, which means the higher expression of Gankyrin protein in cervical precancerous lesion and carcinoma tissues (Table S1). To determine whether the difference of the protein level of Gankyrin between each grades of cervical disease has statistically significance, we compare them as follows: Normal cervical squamous epithelial (NC) *vs* CIN I, NC *vs* CIN II–III, NC *vs* SCC, CIN I *vs* CIN II-III, CIN I *vs* SCC, CIN II-III *vs* SCC. The difference of most of the above group has statistical significance (*p*<0.01), except for NC *vs* CIN I and CIN II–III *vs* SCC (Table S2). In further investigation of the role of Gankyrin in cervical carcinoma, we found that the positive expression rate of Gankyrin had no significant difference between the patients of less than 45 years old and over 45 years old, and neither no significant relationship with histopathological grade and the metastasis of lymph node (*p*>0.05) (Table 1).

**Table 1:** Relationship between clinical pathologic characteristics and expression of Gankyrin in cervical carcinoma tissues.

Factors	Number of patients	Negative	Positive			*P*-value
			+	++	+++	
*Age (year)*						
<45	19	0	0	15	4	0.2973
≥45	21	0	1	11	9	
*Histological grade*						
Low (I)	1	0	0	1	0	0.1112
Intermediate (II)	18	0	1	13	4	
High (III)	21	0	0	13	9	
*Lymph node metastasis*						
3	31	0	1	21	9	0.3405
Yes	9	0	0	5	4	

### 2 The Difference of the Expression of Gankyrin in Paired Cervical Tumor and Carcinoma Adjacent Tissues

**Figure 2:**
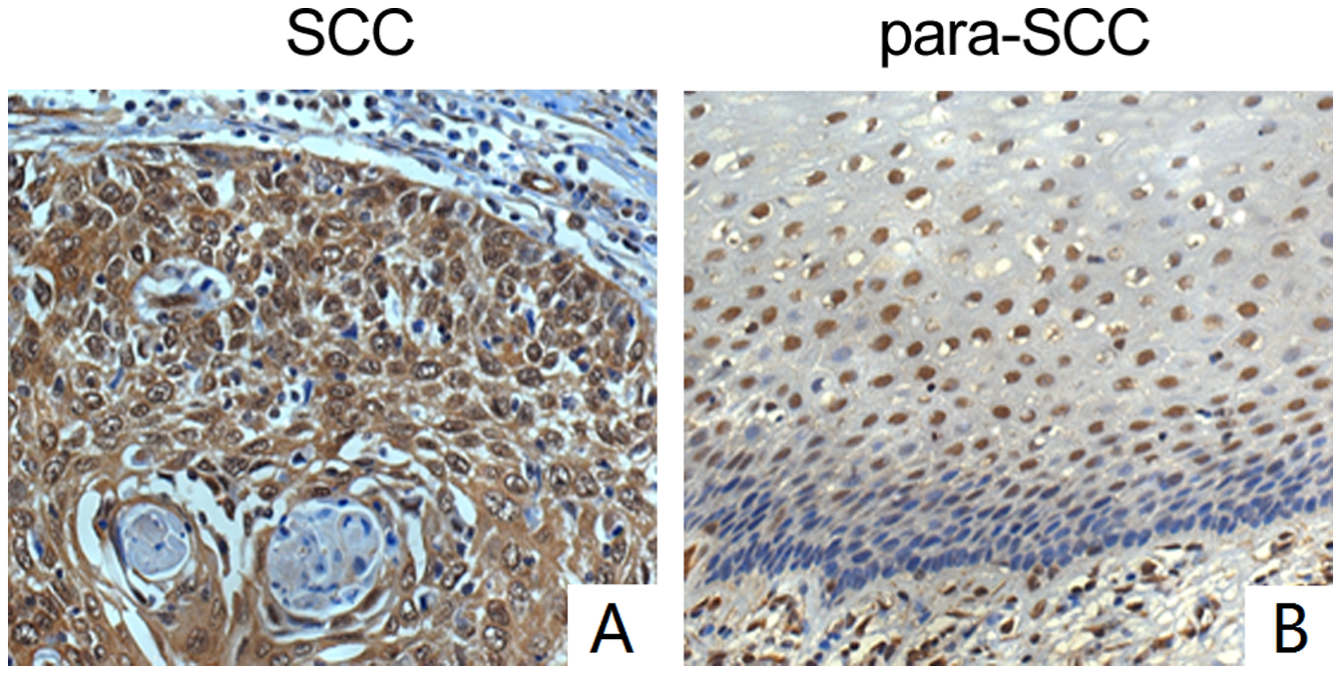
The expression of Gankyrin in 30 pairs of cervical tumor and tumor adjacent tissues by immunohistochemistry staining assay (×200): A. The expression of Gankyrin in cervical carcinoma tissues. B. The expression of Gankyrin in carcinoma adjacent tissues.

To evaluate the potential metastasis of cervical cancer, we analyzed the expression of Gankyrin in 30 pairs of cervical carcinoma tissues and carcinoma adjacent tissues. In these tissues, the positive rate of the expression of Gankyrin is 100% (30/30) in cervical carcinoma tissues, higher than that of carcinoma adjacent tissues (63.3% 19/30), with the value of *p*<0.01 (Figure.2). The staining of Gankyrin in carcinoma adjacent tissues was heterogeneous and weak compared to the abundant and strong expression in carcinoma tissues (Figure S1). In carcinoma adjacent tissues, the lymph node metastasis rate is 0% (0/10) when the nuclei of Gankyrin expression stays positive. However, lymph node metastasis rate turns to be 30% (6/20) when the nuclei of Gankyrin expression stays negative.

### 3 Gankyrin Contributes to the Proliferation in Cervical Carcinoma Cell Lines

**Figure 3:**
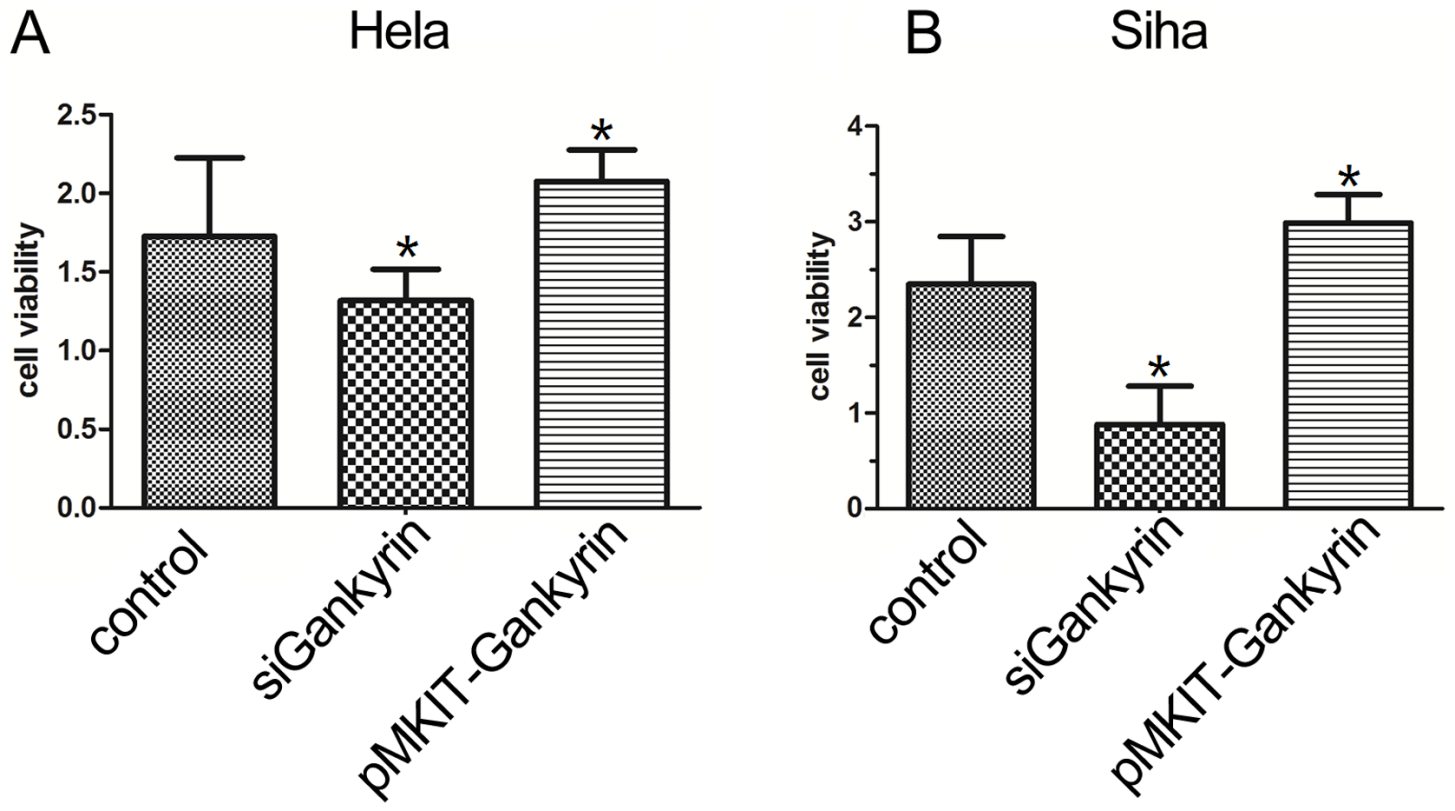
The effects of pMKITneo-hGankyrin plasmid or siGankyrin transfections on cervical carcinoma cells prolifection. The histograms represent cell proliferation at 72h after transfection with pMKITneo-hGankyrin plasmid and siRNA of Gankyrin, respectively. The proliferation of SiHa and HeLa cells transfected with pMKITneo-hGankyrin plasmid was significantly higher than the control groups (*p<0.05). Gankyrin siRNA inhibited proliferation of SiHa and HeLa cells, as compared to the control groups (*p<0.05).

**Figure 4:**
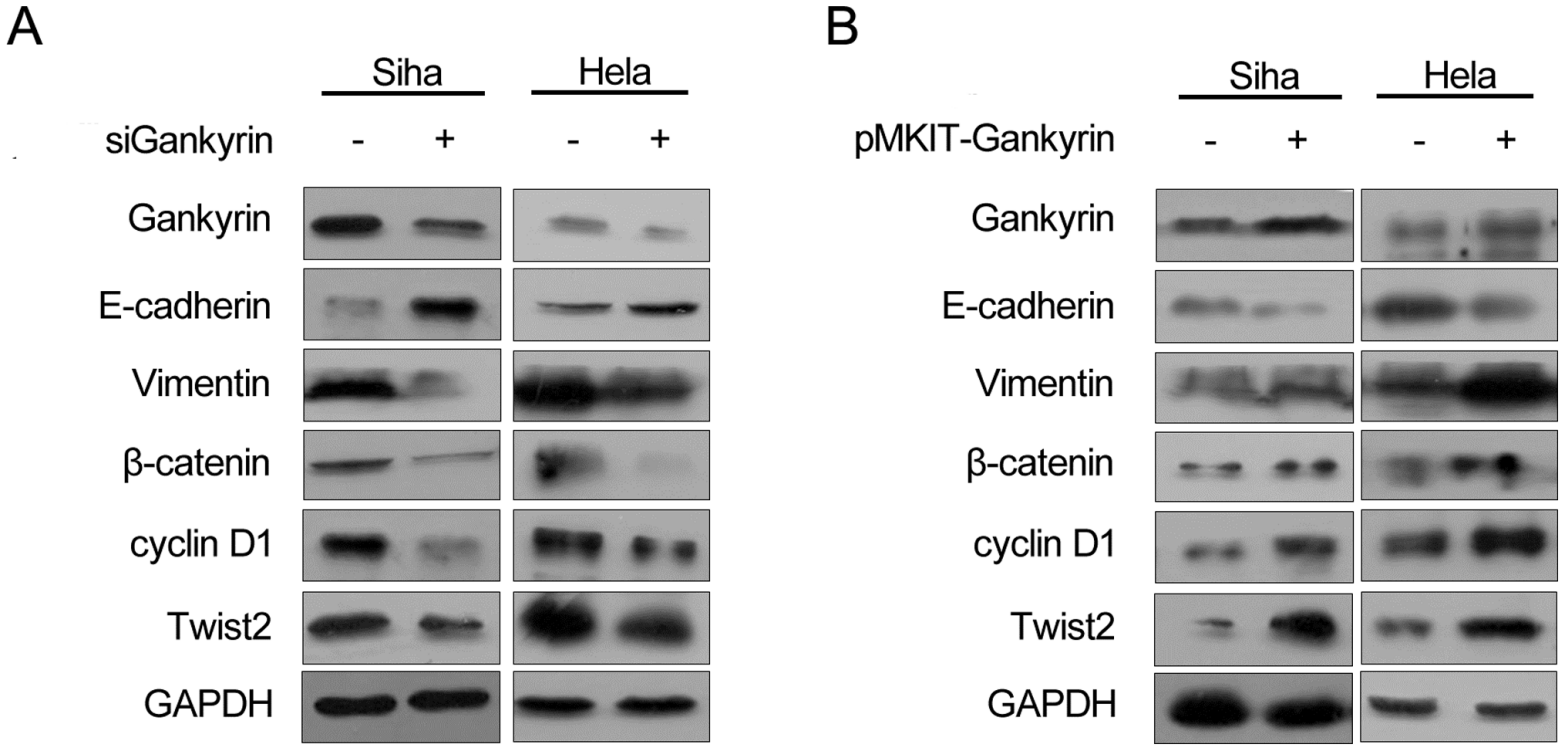
The expression of the hallmarks of EMT (E-cadherin, Vimentin), β-catenin, Twist2 and cyclinD1 after SiHa and HeLa cells were transfected with Gankyrin plasmid or siRNA of Gankyrin. The transfection of Gankyrin could lead to the up-regualtion of β-catenin, Twist2, cyclinD1 and Vimentin, as well as the down-regulation of E-cadherin. While the knock-down of Gankyrin had an opposite effect on SiHa and HeLa cells line.

The roles of Gankyrin on cell growth were investigated by the siRNA-mediated knockdown and pMKITneo-hGankyrin plasmid transfection of Gankyrin in HeLa and SiHa cells. As seen in Figure 3, the CCK-8 assay indicated that pre-treatment with siGankyrin transfection markedly inhibited HeLa and SiHa cells’ proliferation, and Gankyrin’s over-expression led to the opposite results. Meanwhile the expression of cyclinD1 have increased with the transfection of Gankyrin, and the knock-down of Gankyrin has introduced its expression’s reduction (Figure.4).

### 4 Gankyrin Affected Invasion of Cervical Carcinoma Cell Lines

**Figure 5:**
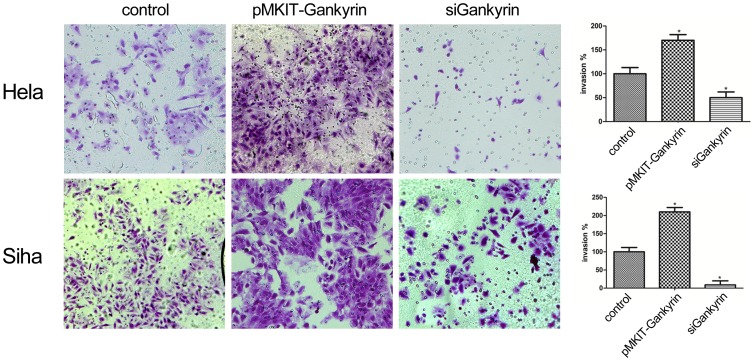
Effect of Gankyrin on invasion of SiHa and HeLa cells line. Microphotographs show representative fields of Giemsa-stained lower membranes of the Boyden chambers; histograms show number of pMKITneo-hGankyrin plasmid and siRNA of Gankyrin treated cells counted by trypan blue exclusion (expressed as % of untreated cells taken as 100). *p<0.05, compared with SiHa-blank group.

To determine the effect of Gankyrin on invasion of cervical carcinoma cells, the two cell lines were transfected with Gankyrin siRNA for 48 hours. It is found that Gankyrin-transfected HeLa and SiHa cells were markedly more invasive than the untreated counterpart with the value of *p*<0.05, and Gankyrin overexpression led to the opposite results (Figure.5). Corresponding to the invasion assay, Gankyrin-transfected cells could also introduce the changes of some epithelial-mesenchymal transition related proteins, such as the up-regulation of Vimentin, Twist2 and β-catenin as well as the down-regulation of E-cadherin. On the contrary, the depletion of Gankyrin resulted in the opposite expression profiles(Figure.4).

## Discussion

The importance of the dual inactivation of both the Rb and p53 pathways for oncogenesis has been demonstrated in an animal model of malignant transformation. ‘High-risk’ HPV E6 oncoprotein down-regulates p53 activity by promoting its degradation through the ubiquitin-dependent proteolytic pathway followed by proteasome-mediated degradation. Meanwhile, HPV E7 oncoprotein is able to regulate the cell cycle by binding to several cellular proteins, including the pocket protein family members, pRb, p107, p130 and the cyclin-dependent kinase inhibitors p21^Cip1^ and p27^Kip1^ [12]. In a previous study, with repeated ankyrin domains, Gankyrin, as an oncoprotein can recruit a MDM2/p53 complex to the 26S proteasome and boost the degradation of p53 with the help of MDM2, thus leading to a tumor-prone phenotype [8]. Meng et al [13] further confirmed that Gankyrin enhances pancreatic cancer cell proliferation via the p53 signaling pathway. It is also reported that oncogenetic activity of Gankyrin interacted with the Rb protein and cyclin-dependent kinase 4/6 (CDK4/6). Additionally, Gankyrin dramatically promoted phosphorylation at specific residues of Rb by CDK4/6 in vivo, while the suppression of Gankyrin could down-regulate cyclin A, cyclin D1 and cyclin E, but up-regulate p27^Kip1^ [8], [13]. As above mentioned, we can obviously notice that the classic carcinogenic mechanisms of Gankyrin strikingly resemble the pathway of HPV oncogenes which serves as the major cause of cervical cancer, thus causing our great attention on the role of Gankyrin in cervical lesions. Then here comes to this, what is the relationship between Gankyrin and cervical disease?

It was well demonstrated that Gankyrin was highly expressed in a variety of tumor tissues such as hepatocellular carcinoma, esophageal squamous cell carcinoma, breast carcinoma and endometrial carcinoma, though its expression was weak in some normal tissues [4]–[7]. In our present study, the increased expression of Gankyrin was also observed in CIN II-III and SCC tissues compared with benign cervical tissues. Especially, the protein level of Gankyrin was notably higher in CIN II-III than that of CIN I. This suggests that Gankyrin, as a specific factor of cervical cancer, also has an application to predict high risk disease.

To evaluate the potential role of Gankyrin in the development of cervical carcinoma, our CCK8 assay demonstrated that knockdown of Gankyrin could induce a dramatic cell viability reduction in cervical carcinoma cell lines, and the transfection of Gankyrin could also introduce the increasing viability of SiHa and HeLa cell lines, similar proliferative patterns were also observed in pancreatic cancer [13], breast cancer [7] and cholangiocarcinoma [14],these results demonstrate that Gankyrin attributes to various cancer cells proliferation. It has been reported that cyclin D1 has gained increasing appreciation as to be strongly involved in the G1-S checkpoint of the cell cycle by affecting the activity of Rb [15]. Our study found that transfection of Gankyrin could lead to the overexpression of cyclin D1 of cervical cells line, which is consistent with the poliferative activity, also indicating that Gankyrin might accelerate cell cycle progression in cervical carcinoma and implying Gankyrin’s role in tumorigenesis and progression of cervical cancer.

In 30 pairs of cervical carcinoma tissues and carcinoma adjacent tissues, the protein level of Gankyrin was also investigated. Although the positive expression rate of Gankyrin in carcinoma tissues had no significant relationship with patients’ age, histopathological grade nor the metastasis of lymph node. Nevertheless, in carcinoma adjacent tissues, we found interestingly that lymph node metastasis rate was 0% (0/10) when nuclei Gankyrin expression stayed positive, however lymph node metastasis rate turned to be 30% (6/20) when nuclei Gankyrin stayed to be negative. Does this mean that Gankyrin nuclear-cytoplasm shift in cervical tissues predict a poor prognosis? It is well acknowledged that nuclear factor-κB (NF-κB) is predominantly found in the cytoplasm in an inactive form, and its activation contributes to the development of metastasis, thus leading to a poor prognosis in many cancers. A higher level of active nuclear-localized NF-κB was observed in the metastatic specimens group [16]. NF-κB was progressively increased from normal cervical tissue, CIN, to cervical cancer, and was also positively associated with stage, histological grade, lymph node metastasis, and invasive interstitial depth [17]. Yao et al [18] found that Gankyrin can enter the nucleus and displace NF-κB from its DNA-binding sites, and then transport NF-κB back to the cytoplasm, thereby carrying out a post-induction repression of NF-κB function. So the Gankyrin nuclear-cytoplasm shift might have critical roles in the process of early metastasis of cervical carcinoma.

Epithelial-mesenchymal transition was implicated in the metastasis of primary tumor, but little study has examined the effects of EMT in the metastasis of cervical carcinoma. It has been reported that knockdown of Gankyrin in colorectal cancer cells impairs the ability of the cells to migrate, invade, and metastasize in vivo via IL-8 pathway [19]. In breast cancer, Gankyrin is highly overexpressed and its expression correlates strongly with lymph node metastasis. Additionally, the reduction of Gankyrin significantly decreases tumor metastasis to lung in animal models [20]. Song et al [21] also found that Gankyrin inhibited tumor growth and metastasis via STAT3/Akt cellular pathway. We have previously reported that Gankyrin plays an essential role in endometrial carcinoma cell proliferation via the PTEN/PI3K/AKT signaling pathway [7]. Further investigation confirmed that PI3K/AKT/HIF-1α signaling activated by Gankyrin could promote Twist1, VEGF, and metalloproteinase 2 expression, and also promote EMT and motility/invasion of tumor cells [22]. Here, we showed that the transfection of Gankyrin markedly up-regulates Vimentin, Twist2, β-catenin and down-regulates E-cadherin in cervical carcinoma cells. However, Gankyrin knockdown led to the opposite results. It has been reported that over-expression of Twist2 was significantly linked to cervical carcinoma metastasis [23]. Mao et al recently found that Twist2 could induce the EMT phenotype including down-regulation of E-cadherin, and upregulation of β-catenin. They speculated that Twist2 might promote the release of β-catenin from the β-catenin/E-cadherin complex through inhibition of E-cadherin [24]. As the hallmarks of EMT, the up-regulation of Vimentin, Twist2, β-catenin and the down-regualation of E-cadherin caused by the transfection of Gankyrin forcefully indicated Gankyrin’s facilitation effect towards EMT of cervical carcinoma. All the above findings suggested that Gankyrin mediated EMT and invasion via multiple signaling pathways.

In conclusion, we found that the protein level of Gankyrin is intimately involved in cervical carcinoma. In carcinoma adjacent tissues, the negative expression of nuclei Gankyrin might indicate lymph node metastasis. Gankyrin may also play a role in cervical carcinogenesis and metastasis. On the basis of these findings, the inviting possibility of targeting Gankyrin might act as part of predictive and therapeutic methods of cervical carcinoma.

## Supporting Information

Figure S1 **The score of Gankyrin expression in SCC tissues and para-SCC (tumor adjacent tissues).** SCC, cervical squamous-cell carcinoma tissues.
(TIF)

Table S1 **The expression of Gankyrin in cervical tissues.** CIN, cervical intraepithelial neoplasia; SCC, cervical squamous cell carcinoma tissues.
(DOCX)

Table S2 **Results of Multiple Comparisons.** CIN,cervical intraepithelial neoplasia; SCC, cervical squamous cell carcinoma tissues.
(DOCX)
