# Supplementary figures and images for: Gankyrin Is Frequently Overexpressed in Cervical High Grade Disease and Is Associated with Cervical Carcinogenesis and Metastasis
Source: PLoS One. 2014 Apr 21;9(4):e95043. doi: 10.1371/journal.pone.0095043 (PMC3994022; doi:10.1371/journal.pone.0095043)

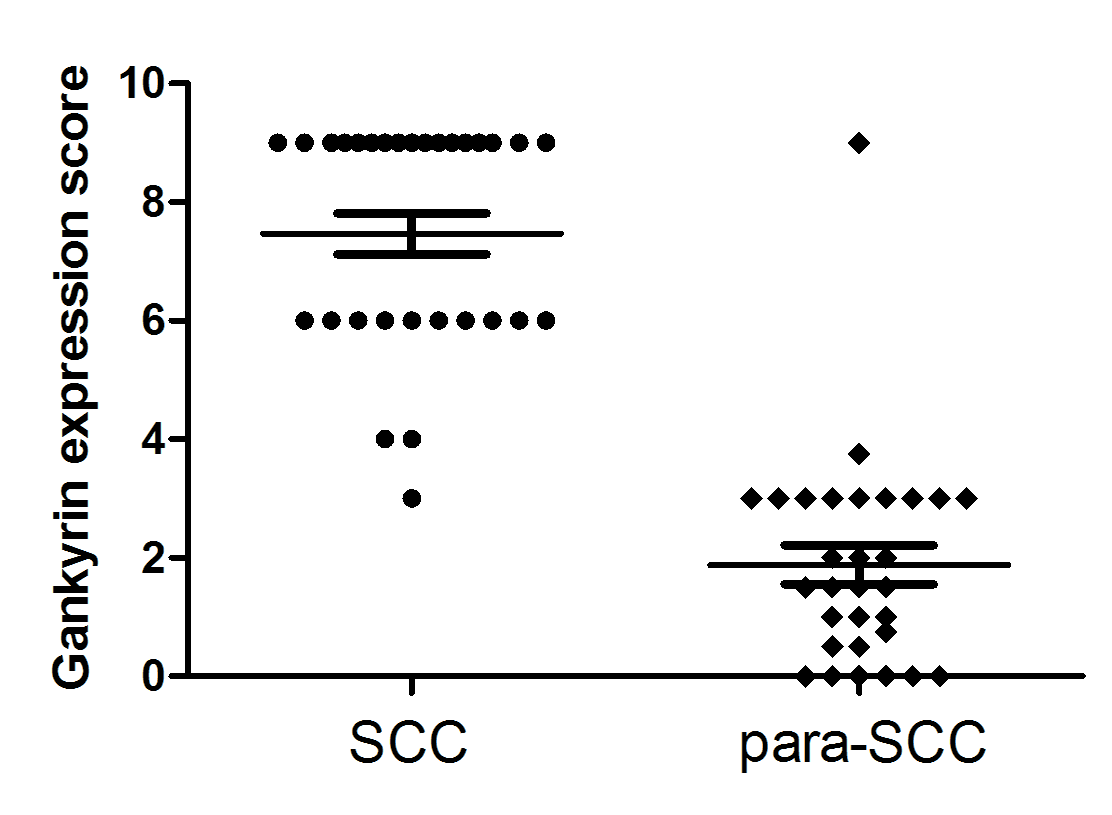

Supplement: Figure S1 — The score of Gankyrin expression in SCC tissues and para-SCC (tumor adjacent tissues). SCC, cervical squamous-cell carcinoma tissues. (TIF) [file pone.0095043.s001.tif]
