# Supplementary material for: Gankyrin Is Frequently Overexpressed in Cervical High Grade Disease and Is Associated with Cervical Carcinogenesis and Metastasis
Source: PLoS One. 2014 Apr 21;9(4):e95043. doi: 10.1371/journal.pone.0095043 (PMC3994022; doi:10.1371/journal.pone.0095043)
Supplement: Table S1 — The expression of Gankyrin in cervical tissues. CIN, cervical intraepithelial neoplasia; SCC, cervical squamous cell carcinoma tissues. (DOCX) [file pone.0095043.s002.docx]

Table S1. The expression of Gankyrin in cervical tissues

| Pathological Type | Index of Gankyrin expression | | | | |
| --- | --- | --- | --- | --- | --- |
|  | — | | + | ++ | +++ |
| Normal | | 1 | 3 | 2 | 0 |
| CIN I | | 1 | 3 | 6 | 1 |
| CIN II-III | | 0 | 2 | 10 | 7 |
| SCC | | 0 | 1 | 26 | 13 |
