# Supplementary material for: Gankyrin Is Frequently Overexpressed in Cervical High Grade Disease and Is Associated with Cervical Carcinogenesis and Metastasis
Source: PLoS One. 2014 Apr 21;9(4):e95043. doi: 10.1371/journal.pone.0095043 (PMC3994022; doi:10.1371/journal.pone.0095043)
Supplement: Table S2 — Results of Multiple Comparisons. CIN,cervical intraepithelial neoplasia; SCC, cervical squamous cell carcinoma tissues. (DOCX) [file pone.0095043.s003.docx]

Table S2. Results of Multiple Comparisons

| Comparisons of tissue groups | *p* value |
| --- | --- |
| Normal *vs* CIN I | 0.2472 |
| Normal *vs* CIN II-III | 0.0041 |
| Normal *vs* SCC | 0.0001 |
| CIN I *vs* CIN II-III | 0.0305 |
| CIN I *vs* SCC | 0.0025 |
| CIN II-III *vs* SCC | 0.8129 |
